# Orientation of Poly(*ε*-caprolactone) in Its Poly(vinyl chloride) Blends Crystallized under Strain: The Role of Strain Rate

**DOI:** 10.3390/ma13245655

**Published:** 2020-12-11

**Authors:** Ruru Wan, Xiaoli Sun, Zhongjie Ren, Huihui Li, Shouke Yan

**Affiliations:** 1 State Key Laboratory of Chemical Resource Engineering, Beijing University of Chemical Technology, Beijing 100029, China; 2018210183@mail.buct.edu.cn (R.W.); xiaolisun@mail.buct.edu.cn (X.S.); renzj@mail.buct.edu.cn (Z.R.); 2 Key Laboratory of Rubber-Plastics, Qingdao University of Science & Technology, Qingdao 266042, China

**Keywords:** poly(ε-caprolactone), poly(vinyl chloride), blend, strain-induced crystallization, crystal orientation

## Abstract

The blends of high and low molecular weights poly(ε-caprolactone) (PCL) with poly(vinyl chloride (PVC) were prepared. The samples before and after the crystallization of PCL were uniaxially stretched to different draw ratios. The orientation features of PCL in a stretched crystalline PCL/PVC blend and crystallized from the amorphous PCL/PVC blends under varied strains were studied by wide-angle X-ray diffraction (WAXD). It was found that a uniaxial stretching of crystalline PCL/PVC blend with high molecular weight PCL results in the *c*-axis orientation along the stretching direction, as is usually done for the PCL bulk sample. For the stretched amorphous PCL/PVC blend samples, the crystallization of high molecular weight PCL in the blends under a draw ratio of λ = 3 with a strain rate of 6 mm/min leads to a ring-fiber orientation. In the samples with draw ratios of λ = 4 and 5, the uniaxial orientation of *a*-, *b*-, and *c*-axes along the strain direction coexist after crystallization of high molecular weight PCL. With a draw ratio of λ = 6, mainly the *b*-axis orientation of high molecular weight PCL is identified. For the low molecular weight PCL, on the contrary, the ring-fiber and *a*-axis orientations coexist under a draw ratio of λ = 3. The *a*-axis orientation decreases with the increase of draw ratio. When the λ reaches 5, only a poorly oriented ring-fiber pattern has been recognized. These results are different from the similar samples stretched at a higher strain rate as reported in the literatures and demonstrate the important role of strain rate on the crystallization behavior of PCL in its blend with PVC under strain.

## 1. Introduction

The multiscale structure control of crystalline polymers is of great importance for regulating their macroscale properties and developing high-performance polymeric materials. It was found that melt crystallization from oriented chains exhibited a great effect on the crystal structure and crystalline morphology and therefore, it has been investigated extensively. For example, the crystallization of isotactic polypropylene (iPP) after pulling the fiber embedded in its melt can induce oriented β crystallization [1,2,3,4,5]. Similar results have also been obtained through the melt recrystallization of highly oriented iPP fibers by controlled melting status, reflecting the different chain orientation in the melt caused by varied chain relaxation [6,7,8,9,10]. This stimulates the extensive studies of crystallization from oriented (amorphous) chains for different polymeric materials, including individual polymers and polymer blends. The studies on the crystallization from elongated chains of individual polymers demonstrated the formation of a uniaxial (or fiber) orientation of them with the *c*-axis aligned in the stretching direction, while the *a-* and *b*-axes were arranged randomly about the *c*-axis [11,12,13,14,15,16,17,18]. However, the orientation of miscible crystalline/crystalline polymer blends crystallized under strain can be quite different from the uniaxial fiber orientation. For instance, the crystallization of poly(1,4-butylene succinate) (PBS) in its miscible blends with poly(vinylidene fluoride) (PVDF, a polymorphic polymer [19]) under strain results in a biaxial orientation of PBS with *b*- and *c*-axes in the film plane and the *b*-axis along the stretching direction [20]. On the other hand, the crystallization of poly[(R)-3-hydroxybutyrate] (PHB) in its miscible blends with PVDF under strain causes an *a*-axis orientation of PHB along the stretching direction [21]. For the crystalline/crystalline polymer blends, the crystal structure and orientation of later crystallized components can be controlled by epitaxy or transcrystallization at the surface of early formed crystals [22,23,24,25,26,27,28,29,30,31,32,33,34,35,36,37], which may not reflect the effect of strain and the blending component on the later crystallized component correctly. It should be pointed out that soft epitaxy can hardly take place in crystalline/amorphous polymer blends, since it is not a common phenomenon [38,39]. It has been confirmed that a soft epitaxy of crystalline polymer does not happen even on oriented films of amorphous materials [40]. In this sense, the miscible crystalline/amorphous binary polymer blends can rule out the epitaxial effect. Thereby, the different structures of this kind of polymer blend produced under strain can illustrate the real influence of strain and other components on the crystallization behavior and result in a unique supermolecular structure of the crystalline component. Therefore, the crystallization and orientation features of the crystalline component in the miscible crystalline/amorphous polymer blends under strain have attracted much attention, and studies show that the crystallization of miscible crystalline/amorphous binary polymer blends under strain can generate quite complicated orientation, crystalline texture, and morphology of the crystalline component [41,42,43,44,45].

For crystalline/amorphous blends, poly(*ε*-caprolactone) (PCL) has been mostly chosen as the crystalline component, since it exhibits only one crystalline modification with rather simple crystal structure and good miscibility with many amorphous polymers [46,47,48,49,50,51,52]. Taking the blend of PCL with amorphous poly(vinyl chloride) (PVC) as an example, systematic studies show that the PCL crystals in the blends can exhibit uniaxial orientations with *a*-, *b*-, or *c*-axis in the stretching direction, as well as a so-called ring-fiber orientation with the *c*-axis oriented perpendicular to the drawing direction, while the a-axis and b-axis are randomly distributed in the plane perpendicular to the c-axis [50,51,52]. Prud’homme et al. [50] found that the crystallization of PCL in PCL/PVC blends under strain leads to the orientation of the *c*-axis perpendicular to the strain direction in most conditions. When stretching the blend films at a strain rate of 20 mm/min immediately after cooling down to room temperature, the ring-fiber orientation is obtained at low draw ratios and/or low PVC contents, while an *a*-axis orientation along the stretching direction together with the dominated ring-fiber orientation are observed with the increasing draw ratio and PVC content [51]. In both cases, the *c*-axis of PCL crystals is characterized by a perpendicular alignment with respect to the stretching direction. A parallel alignment of the PCL chains along the stretching direction is also observed under a high draw ratio, e.g., λ > 7, at a high strain rate of 80 mm/min [50]. Moreover, the molecular weight of the PCL shows an also apparent effect on the crystal orientation of PCL in the blends [52]. It was found that under a draw ratio of λ = 4 at a strain rate of 20 mm/min, PCL with a number average weight (*M_n_*) of 125k exhibits a regular fiber orientation with the *c*-axis aligned along the stretching direction. However, while the PCL with *M_n_* = 13k shows dominantly a ring-fiber orientation, the PCL with *M_n_* = 21k achieves a *b*-axis orientation.

From the above description, it is clear that the orientation of PCL in its blends with PVC depends remarkably on the orientation status of molecular chains in the amorphous state. It is generally accepted that a high strain rate and low temperature means less chain relaxation during the stretching and, thereby, a greater chain orientation. Taking this into account, a lower strain rate and higher temperature will reduce the chain orientation and change the orientation status of the PCL and PVC chains in the amorphous blends. Consequently, different structures of PCL crystallized in the blends under strain are expected. Therefore, the orientation structure of PCL with different molecular weights in the PCL/PVC (70/30) blend crystallized under varied strains at a much slow strain rate of only 6 mm/min and higher temperature of 40 °C was studied in this work. The obtained results are helpful for a further understanding of the underlying mechanism of the different orientation of PCL in its blends with PVC crystallized under strain.

## 2. Experimental

### 2.1. Materials

Two kinds of PCL with low (*M_n_* = 1.75 × 10^4^ g/moL, *Đ* = 1.44) and high (*M_n_* = 9.29 × 10^4^ g/moL, *Đ* = 1.73) molecular weight, denoted as L-PCL and H-PCL, respectively, were supplied by Aldrich Chemical Co., Saint Luis, MO, USA. The PVC with *M_n_* = 9.90 × 10^4^ g/moL and *Đ* = 1.35 was also purchased from Aldrich Chemical Co., Saint Luis, MO, USA. The tetrahydrofuran (THF) solvent was purchased from Tongguang Fine Chemical Co. Ltd., Beijing, China and used without further purification.

### 2.2. Sample Preparation

**Figure 1:**
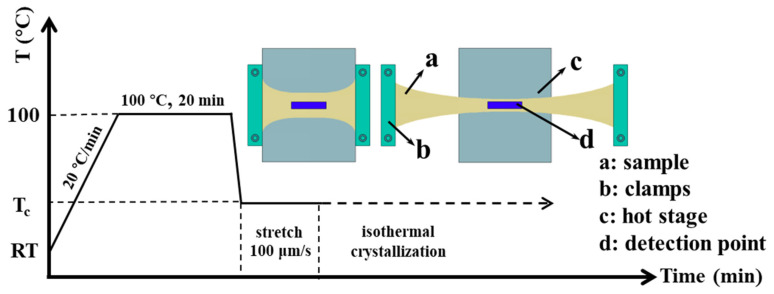
Schematic illustration of the experimental procedure.

PCL/PVC (70/30, weight ratio) blends were prepared by dissolving them together in THF. Films of PCL/PVC blends were prepared by casting their 5 wt % THF solutions at room temperature and dried for 48 h to eliminate the residual solvent. The thickness of the obtained films is ca. 100 μm as measured by a Dektak Profilometer (Bruker Co. Ltd., Karlsruhe, Germany). As schematically presented in Figure 1, the PCL/PVC thin films were first heat-treated at 100 °C for 20 min to erase the previous thermal history; then, they were cooled rapidly down to 40 °C and stretched uniaxially to a certain draw ratio of λ. The PCL in the blends was finally allowed to crystallize under strain isothermally at 40 °C. The draw ratio λ is defined as λ = *l*/*l_0_*, where *l* and *l_0_* are the film lengths after and before stretching, respectively. The stretching of the sample was performed by using a micro tensile machine (Linkam TST350, Linkam Scientific Instruments Ltd., Tadworth, UK) at a speed of 6 mm/min, which is much slower than the used strain rate in other literature [50,51,52].

### 2.3. Wide-Angle X-ray Diffraction (WAXD)

Wide-angle X-ray diffraction (WAXD) measurements were used to monitor the crystal orientation of PCL in miscible PCL/PVC blends under the different draw ratio at 1W2A beam line in Beijing Synchrotron Radiation Facility with wavelength of 0.154 nm. Two-dimension (2D) WAXD patterns are collected with a Mar165 CCD detector (2048 × 2048 pixels with pixel size of 79 μm) and analyzed with Fit 2D software (European Synchrotron Radiation Facility, Grenoble, France). The exposure time for each pattern is 20 s.

## 3. Results

### 3.1. Structure Evolution of Crystalline PCL in Its Blends with PVC under Strain

**Figure 2:**
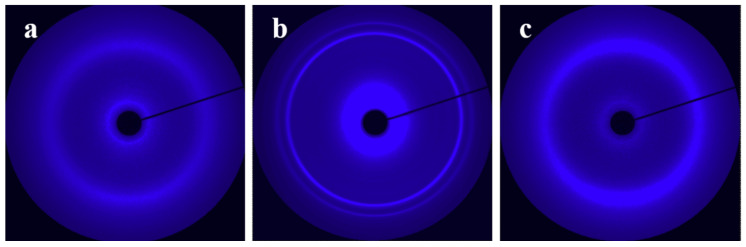
Wide-angle X-ray diffraction (WAXD) patterns of the high molecular weight poly(ε-caprolactone) H-PCL/poly(vinyl chloride) (PVC) blends cooled from 100 °C down to and kept at 40 °C for (**a**) 0 h and (**b**) 40 h. Part (**c**) was recorded after cooling from 100 °C down to 40 °C and then stretched immediately with a draw ratio of λ = 6 at a strain rate of 6 mm/min. The draw direction is horizontal.

Figure 2a shows the WAXD pattern of an H-PCL/PVC blend film recorded immediately after the sample cooled from 100 down to 40 °C. The WAXD pattern shows only an amorphous halo, demonstrating the suppression of H-PCL crystallization by PVC in the cooling process from 100 to 40 °C. This is in good accordance with the literature reports and has even been observed in the PCL/PVC blend on the oriented polyethylene substrate, which is confirmed to exhibit high nucleation ability toward PCL [29,37,53]. Actually, the crystallization of H-PCL in the H-PCL/PVC can take place at 40 °C with prolonged time. As presented in Figure 2b, two diffraction rings appear in the WAXD pattern after keeping the sample at 40 °C for 40 h. These two diffraction rings can be accounted for by the orthorhombic unit cell of PCL with dimensions *a* = 0.748 nm, *b* = 0.498 nm, and *c* = 1.729 nm as the (110) and (200) reflections [51]. It is well documented that strain can tremendously enhance the crystallization rate of polymers. To check whether the strain can promote the crystallization of H-PCL in the H-PCL/PVC blend, a blend film cooled first quickly from 100 down to 40 °C and subsequently stretched with a draw ratio of λ = 6 at a strain rate of 6 mm/min was studied by WAXD. To our surprise, as shown in Figure 2c, the WAXD pattern shows still only the amorphous halo, implying that the crystallization of H-PCL in the H-PCL/PVC blend does not take place during stretching immediately after cooling the thin film down to 40 °C as well. Similar results have also been obtained for the L-PCL/PVC system as presented in the Figure S1 in the electronic Supplementary Information (ESI). The delayed crystallization of PCL in its blends with PVC provides the opportunity for comparing the orientation of PCL in its amorphous blends with PVC crystallized under strain and the structure evolution of crystalline PCL in the PCL/PVC blends during deformation.

**Figure 3:**
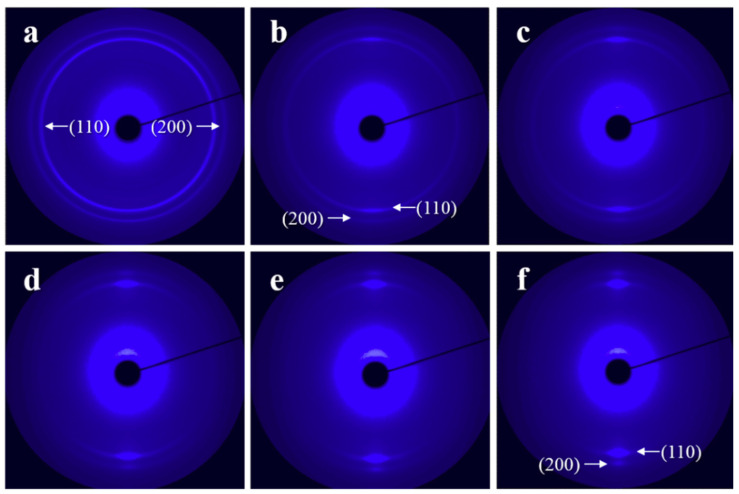
WAXD patterns of crystalline H-PCL/PVC (70/30) blend films drawn with a strain rate of 6 mm/min at room temperature to various draw ratios. (**a**) *λ* = 1, (**b**) *λ* = 2, (**c**) *λ* = 3, (**d**) *λ* = 4, (**e**) *λ* = 5, and (**f**) *λ* = 6. The draw direction is horizontal.

Figure 3 presents the structure evolution of H-PCL crystals in the H-PCL/PVC blends during deformation. As seen from Figure 3a, the original sample exhibits non-oriented PCL crystals, which produce strong (110) and weak (200) diffraction rings with even intensity. Stretching the sample results in a gradually orientation of the PCL crystals. With a draw ratio of λ = 2, the strong (110) and weak (200) diffractions exhibit already reflection intensity maximum on the meridian direction. The intensity of these diffractions on the meridian direction get stronger with further increase of the draw ratio. At a draw ratio of λ = 6, sharp and well-defined (110) and (200) diffraction spots can be observed on the meridian direction (see Figure 3f), indicating a high orientation of the H-PCL crystals. The arrangement of these diffractions in the direction vertical to the stretching direction demonstrates a *c*-axis orientation of the H-PCL crystals along the drawing direction, while the *a*- and *b*-axes are oriented randomly in the plane perpendicular to the strain. This is similar to the orientation achieved by stretching the crystalline PCL in the solid state through transition from spherulites into the microfibrils [54].

### 3.2. Crystallization of H-PCL in H-PCL/PVC Blend under Strain

**Figure 4:**
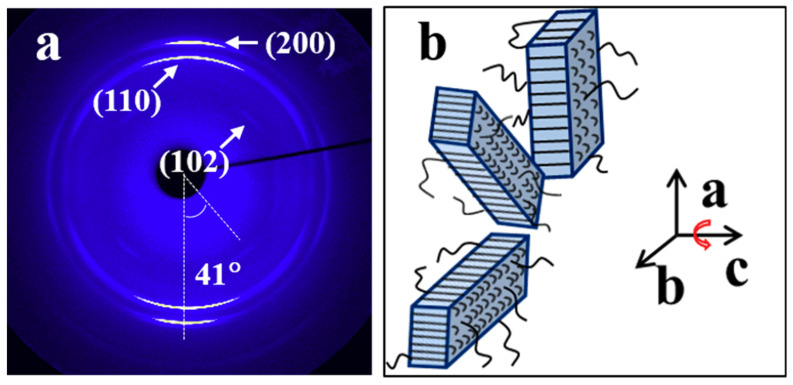
(**a**) A WAXD pattern with main reflections being indexed of an H-PCL/PVC blend film cooled from 100 °C down to 40 °C, immediately stretched to a draw ratio of λ = 3 at a strain rate of 6 mm/min and then crystallized at 40 °C under strain for 12 h. (**b**) A sketch illustrating the ring-fiber orientation of H-PCL crystals in the blend. The stretching direction is vertical.

Figure 4a shows the WAXD pattern with main reflections being indexed of an H-PCL/PVC blend film cooled from 100 °C down to 40 °C, immediately stretched to a draw ratio of λ = 3, and then crystallized at 40 °C under strain for 12 h. Its corresponding azimuthal profiles can be found in Figure S2 in ESI. Strong (200) and (110) diffractions with maximum intensity in the stretching direction can be clearly seen. With close inspection, very weak (102) diffractions, which are inclined ca. ±41° with respect of the (200) diffractions, can be identified. According to this X-ray diffraction result, it is concluded that by crystallizing the H-PCL in its blend with PVC under a strain of λ = 3 at 40 °C, a *c*-axis uniaxial orientation is produced, i.e., a unique *c*-axis orientation but a random orientation of *a*- and *b*-axes in the plane perpendicular to the *c*-axis. However, the thus produced *c*-axis orientation is different from the one shown in Figure 3, where the *c*-axis is aligned along the stretching direction. Now, the *c*-axis of H-PCL is aligned perpendicular to the drawing direction. This kind of orientation has been referred to as ring-fiber orientation, as schematically presented in Figure 4b, by Kakudo and Kasai [55] and also observed for a PCL/PVC (80/20) blend crystallized at room temperature under a draw ratio of λ = 3.6 at a strain rate of 20 mm/min [51]. For the stretched blends, the vertical *c*-axis orientation with respect to the strain direction is unusual, since the crystallization of stretched PCL is expected to start from the chains oriented by stretching, similar to the structure of melt-draw induced crystallization of polymers, where the *c*-axis is always aligned along the stretching direction [11,12,13,14,15,16]. This demonstrates the influence of PVC on the crystal orientation of the PCL.

**Figure 5:**
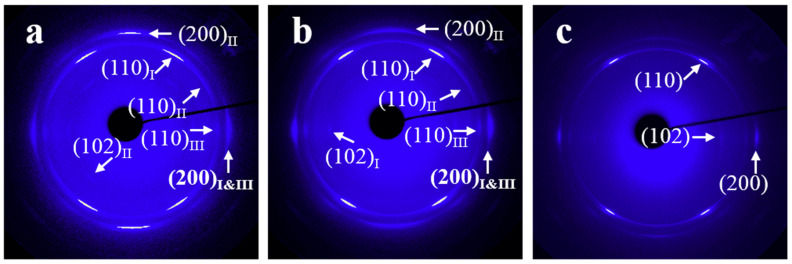
WAXD patterns with main reflections being indexed of a H-PCL/PVC blend films cooled from 100 °C down to 40 °C, immediately stretched to different draw ratios, and then crystallized at 40 °C under strain for varied times. (**a**) λ = 4, *t_c_* = 10 h; (**b**) λ = 5, *t_c_* = 8 h; (**c**) λ = 6, *t_c_* = 5 h. The stretching direction is vertical.

Figure 5 shows the WAXD patterns of H-PCL/PVC blend films crystallized at 40 °C under different draw ratios for varied times (*t_c_*). Figure S3 in the ESI shows their corresponding azimuthal profiles. From Figure 5a, it can be seen that the crystal orientation of H-PCL in the H-PCL/PVC blend crystallized under a strain of λ = 4 for *t_c_* = 10 h is very complicated. As indexed in the picture by the subscript of I, II, and III, there are totally three different orientations of the H-PCL. The first one shows the strong (110) diffractions (described as (110)_I_ in Figure 5a) at the positions ca. ±33° inclined from the stretching direction. This implies an *ab* plane with the *b*-axis aligned in the stretching direction, whereas the *a*-axis is oriented perpendicular to the stretching direction. In this case, these crystals contribute also the (200) diffractions in the positions vertical to the stretching direction. Moreover, weak (102) diffractions in the equator direction can also be recognized with close inspection. This suggests a random rotation of *a*- and *c*-axes about the *b*-axis. In other words, a *b*-axis orientation of this part of the H-PCL crystals has been achieved. The second one exhibits strong (200) diffractions, i.e., (200)_II_ in the direction of stretching. The weak (110)_II_ diffractions located at the positions ca. ±57° away from the stretching direction are associated to this part of oriented crystals. Considering that the scattering factor of the (110) lattice plane is much higher than the (200) one, the reduced intensity of the (110)_II_ diffractions suggests an *a*-axis orientation in the direction of stretching and a random rotation of *b*- and *c*-axes about the *a*-axis. In addition, the appearance of weak (102)_II_ inclined ca. ±41° away from the *a*-axis direction supports an *a*-axis orientation of these crystals. The third one contributes weak (110)_III_ diffractions in the direction perpendicular to the stretching direction. This may suggest a *c*-axis orientation along the stretching direction. In this way, these crystals should also contribute (200) reflections in the equator direction, which overlap with the (200) diffractions contributed by the uniaxially *b*-axis oriented crystals. Therefore, the (200) diffractions in the vertical direction with respect to strain have indexed as (200)_I&III_ in Figure 5a. When crystallizing the H-PCL/PVC under a strain of λ = 5 for 8 h, as shown in Figure 5b, essentially the same diffraction pattern as that shown in Figure 5a was obtained. However, the orientation extent of the H-PCL crystals increased to some extent. Moreover, the *a*-axis orientation along the stretching direction reduced very much (compare the intensity of (200)_II_ diffractions on the strain direction shown in Figure 5a,b). This also results in the disappearance of the (102)_II_ diffraction ±41° away from the stretching direction in Figure 5b. At the same time, weak (102)_I_ diffractions in the equator direction can be more clearly seen, demonstrating an increased *b*-axis orientation along stretching direction. With further increase of the draw ratio to λ = 6, except for the increased orientation of H-PCL crystals as reflected by the sharp diffraction spots, mainly the *b*-axis orientation remains.

### 3.3. Crystallization of L-PCL in L-PCL/PVC Blend under Strain

The above experimental results reveal the draw-ratio-dependent orientation of H-PCL crystals in the H-PCL/PVC blends grown under strain. To check the influence of molecular weight on the orientation behavior of PCL in its blends with PVC during crystallization under strain, the structure of low molecular weight PCL in the L-PCL/PVC blends produced under different strains was studied. It was found that the stretching ability of crystalline L-PCL/PVC is much poorer than the H-PCL/PVC. As presented in Figure S4, a maximum strain of only about λ = 1.7 can be achieved. The limited deformation of the sample does not produce obvious orientation of the sample as judged from the WAXD patterns, and therefore, we will not discuss it anymore here. The stretchability of the amorphous L-PCL/PVC is much better than its crystalline counterpart, even though it is poorer than the H-PCL/PVC one. It can reach a maximum draw ratio of λ = 5.

**Figure 6:**
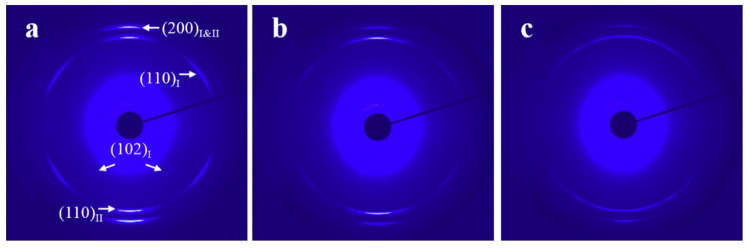
WAXD patterns of L-PCL/PVC blend films crystallized at 40 °C under various draw ratios for different times. (**a**) *λ* = 3, *t_c_* = 4 h; (**b**) *λ* = 4, *t_c_* = 2.5 h; and (**c**) *λ* = 5, *t_c_* = 2 h. The drawing direction is vertical.

Figure 6 and Figure S5 show the WAXD patterns and corresponding azimuthal profiles of L-PCL/PVC blend films crystallized at 40 °C under different draw ratios for varied times, respectively. The diffraction pattern shown in Figure 6a with a draw ratio of λ = 3 is composed of two sets of oriented crystals. The strong (200) diffractions located in the stretching direction are contributed by both sets of the oriented crystals and consequently indicated as (200)_I&II_. These (200) diffractions together with the (110)_I_ diffractions have a moderate intensity, and the very weak (102)_I_ diffractions are contributed by crystals exhibiting an *a*-axis orientation along the stretching direction, while the *b*- and *c*-axes are randomly oriented in the plane perpendicular to the *a*-axis. The other set oriented L-PCL crystals contribute both the strong (110)_II_ and (200)_I&II_ reflections in the drawing direction, demonstrating a ring-fiber orientation with the *c*-axis orientation in the direction perpendicular to the strain. This is different from the H-PCL/PVC with λ = 3, where only the ring-fiber orientation as illustrated in Figure 4 is identified. With increase of draw ratio, the *a*-axis orientation reduces gradually and disappears completely at a draw ratio of λ = 5, leading to a solely ring-fiber orientation (Figure 6c). Moreover, the crystallinity and orientation extent of the sample crystallized under λ = 5 is obviously decreased as judged from the broader (110) diffraction arcs with reduced intensity. These results reveal clearly the influence of molecular weight on the PCL orientation in its blends with PVC in the following aspects. First, the toughness of the sample decreases tremendously, especially for the crystalline one, reflecting the reduced entanglement due to the shortened chain length. Second, the orientated structure of the L-PCL crystals in the L-PCL/PVC blends grown under various draw ratios is different from its high molecular counterpart.

## 4. Discussion

According to the obtained results, several aspects can be discussed here. First, it is now clear that the orientation of H-PCL in the crystalline H-PCL/PVC sample during deformation differs remarkably from the H-PCL crystals grown from amorphous H-PCL/PVC samples under strain. While the deformed crystalline sample exhibits only a *c*-axis orientation along the stretching direction, the crystallization from amorphous samples under strain produces complicated orientations depending on the draw ratio and molecular weight. This can be explained in the following way. Even though the PCL exhibits excellent miscibility with PVC over the whole range of composition, the crystallization of PCL results unambiguously in the phase segregation, since the amorphous PVC cannot pack into the crystal lattice of the PCL. This will lead to the non-crystallizable PVC dispersing in the amorphous regions of PCL. In this case, the orientation of crystalline PCL in the blends during deformation will be less affected by the PVC in the amorphous regions. Therefore, the *c*-axis orientation, or a fiber orientation frequently used in the literature, is always produced during deformation of the samples. On the other hand, blending PCL with PVC hinders the crystallization of PCL remarkably. As a result, the PCL does not crystallize directly after cooling from 100 °C down to 40 °C as well as immediately stretched to different draw ratios (see Figure 2 and Figure S1 in the ESI). This produces a homogeneously distributed amorphous PCL/PVC blend at a molecular scale. Consequently, the PCL and PVC chains or chain segments will be simultaneously and synergistically deformed during stretching. This results in an enhanced influence of PVC on the crystallization of PCL and leads to the complicated orientation behavior of PCL crystals, which will be discussed in the following section.

According to the above described experimental results, in total, four different uniaxial orientations have been recognized for PCL crystals grown in the blends with PVC under strain. They are the uniaxial orientation of *a*-, *b*-, and/or *c*-axis along the strain direction, as well as the so-called ring-fiber orientation. The *c*-axis orientation along the stretching direction is most easily understood. It is caused by highly oriented chains or chain segments, which serve as row nuclei for the subsequent growth of PCL chain fold lamellar crystals and lead to the chain fold lamellae oriented perpendicular to the stretching direction. The *b*-axis orientation along the strain direction under strain is best understood up to date and has been attributed to the result of confined crystallization of PCL in the blends with PVC. It was found that the orientation of amorphous PVC takes place rapidly during stretching [48]. In this case, it makes sense that both PCL and PVC chains or chain segments in amorphous will be oriented under strain. Considering that the PVC has a high glass transition temperature of around 89 °C, which is much higher than the used stretching temperature of 40 °C, the oriented PVC will essentially remain unchanged during the crystallization of PCL at 40 °C. On the other hand, the study on the homogenously oriented poly(L-lactide) (PLLA)/poly(vinyl acetate) blends has found that the blend undergoes first a microphase separation prior to the crystallization of PLLA [56]. This may happen also for the PCL/PVC system. Consequently, it is conjectured that the phase separation under strain will lead to the formation of elongated microdomains of both PCL and PVC. Thus, the crystallization of PCL will occur within the micro-gaps formed by elongated PVC domains, which provides a confined environment for the molten PCL in the subsequent crystallization process. The dimensions of the PVC micro-gaps should depend on the draw ratio. They will be elongated and narrowed with the increasing draw ratio. Therefore, the confined crystallization of PCL in its blend with PVC under a high draw ratio produces solely a *b*-axis orientation (Figure 5c), since the *b*-axis is the fastest growth direction of the PCL crystal. This has also been found for the PCL crystallized in smaller nanocylinders with a diameter of 13.0 nm [57]. The ring-fiber orientation has been attributed to the crystallization of amorphous PCL chains in the oriented state caused by chain relaxation and longitudinal retraction through intramolecular nucleation, which results in an alignment of the *c*-axis perpendicular to the stretching direction. In this case, the random orientation of *a*- and *b*-axes in the plane perpendicular to the *c*-axis suggests a comparable length scale of micro-gaps composed of PVC along and perpendicular to the stretching direction and, thereby, the growth of the PCL crystals along its fastest *b*-axis direction can propagate randomly in the plane perpendicular to *c*-axis, results in a ring-fiber orientation. This is probably the reason for the crystallization of PCL with ring-fiber orientation under low strain, since a larger length scale of micro-gaps along stretching than vertical to the stretching direction is expected with an increased draw ratio. For the *a*-axis orientation, a rational explanation is still lacking. Considering that the folding of PCL chain during crystallization is along its *a*-axis direction, Prud’Homme et al. [51] suggest that the *a*-axis orientation is most likely related to the nucleation of retracted chains and growth of crystals through folding along the original chain direction cause by strain. Actually, there may be another possibility for the *a*-axis orientation. It has been confirmed that cavitation can take place during the tensile deformation of polymers [58,59]. The cylinder-shaped cavities could be oriented with a long axis perpendicular to the stretching direction under certain strain. In the present case, taking the high T_g_ and low content of PVC into account, there may be perpendicularly arranged cylinder-shaped cavities that are filled by high mobility PCL chains through plastic flow. If this is true, the combination of intramolecular nucleation and crystal growth of PCL along its fastest *b*-axis will produce the *a*-axis orientation.

In the present work, greater emphasis was laid on the influence of strain rate toward the orientation of PCL crystals grown in its blends with PVC under different strains. To this end, an extremely low strain rate was used with respect to those used in the literature. Indeed, our results show some differences compared to the literature reports. First, it is reported by Hubbell and Cooper. [49] that by stretching the PCL/PVC (70/30) blend films at a strain rate of 20 mm/min immediately after cooling down to room temperature, ring-fiber orientation is always observed under draw ratios from 2 to 7. The *a*-axis orientation is obtained only when the draw ratio is larger than 2.7 with content remains almost unchanged in the draw ratio range from 2.7 to 7. We found on the contrary that the ring-fiber and *a*-axis orientations for the L-PCL/PVC blend coexist only at draw ratios less than λ = 5 and the *a*-axis orientation decreases with the increasing draw ratio (see Figure 6a,b). This is not influenced by molecular weight, since the molecular weights of PCL used in this work and the literature are similar (17.5k and 18.0k, respectively). It should actually be related to the different orientation status and phase structure caused by stretching at different strain rates and the temperatures used for the subsequent crystallization of PCL. In our case, stretching at a high temperature of 40 °C with a very low strain rate of only 6 mm/min means an easier and faster chain relaxation during the stretching and, thereby, a less chain orientation. Taking this into account, our results may suggest that a higher chain orientation does not favor the crystallization of PCL in an *a*-axis orientation. This is of course only a hypothesis, since the slow deformation will certainly also influence the orientation status of PVC and the microphase structure of the blends as well. In essence, the different crystallization behavior should be associated to the combined and synergistic effects of the microphase structure, chain orientation of both polymers, crystallization kinetics, etc. Second, it has been reported by Coleman et al. [48] that *c*-axis orientation can be achieved with a high strain rate of 230 mm/min at a draw ratio of λ = 4.5, while no *c*-axis orientation has been observed with a strain rate of 35 mm/min even at a high draw ratio above 5. This is also different from our results on the H-PCL/PVC. We found that all orientations of *a*-, *b*-, as well as the *c*-axis along the strain direction appear at a low strain of λ = 4. However, at a high draw ratio of λ = 6, only the *b*-axis orientation remains. This may imply that deformation at a low rate enhances the relaxation of PCL and at the same time facilities the formation of narrow PVC gaps. In this way, the *c*-axis orientation does not take place, while the crystallization of relaxed PCL chains in the narrow gaps of PVC produces predominantly a *b*-axis orientation. In summary, there are many factors that influence the crystallization behavior of PCL in its PVC blends under strain. It is still far from a full understanding of the sophisticated orientation behavior of PCL in its PVC blends grown under strain. Therefore, further detailed studies are clearly warranted.

## 5. Conclusions

The blends of high and low molecular weights of PCL with PVC were prepared. It was found that blending PVC with PCL deceased the crystallization ability of PCL. Therefore, the orientation features of PCL in a stretched crystalline PCL/PVC blend and crystallized from the stretched amorphous PCL/PVC blends under varied strains were compared. The results show that stretching the crystalline H-PCL/PVC blends results in a *c*-axis orientation along the stretching direction of the high molecular weight PCL. Due to the limited stretchability, the low molecular weight PCL in the L-PCL/PVC blends does not orient obviously. On the contrary, the orientation of the PCL crystals grown in the stretched amorphous PCL/PVC blends under strain is quite complicated, depending on the molecular weight of PCL, the draw ratio, and the strain rate. In total, four different orientations of PCL grown under different draw ratios at a strain rate of 6 mm/min have been identified. They are the uniaxial orientations of the *a*-, *b*-, or/and *c*-axis along the strain direction as well as the so-called ring-fiber orientation. The acquirement condition of each orientation is different from the reported results. For example, it has been reported that ring-fiber and *a*-axis orientations for the PCL/PVC (70/30) blend are obtained at a strain rate of 20 mm/min with draw ratios from 2 and 2.7 to 7, respectively. In the present work, the ring-fiber and *a*-axis orientations of PCL having a similar molecular weight in the L-PCL/PVC (70/30) coexist only with a draw ratio less than 5. Moreover, it has been claimed that the *c*-axis orientation along the stretching direction can be achieved under a high draw ratio of λ = 4.5 at a high strain rate of 230 mm/min. In the H-PCL/PVC case, all uniaxial orientations of the *a*-, *b*-, as well as *c*-axis along the strain direction appear at a strain of λ = 4, while only the *b*-axis orientation along the strain direction remains with a draw ratio of λ = 6.

These results suggest the existence of many factors that influence the crystallization behavior of PCL in its PVC blends under strain. For a better understanding of the sophisticated orientation behavior of PCL in its PVC blends grown under strain, further detailed studies are warranted.

## Supplementary Materials

The following are available online https://www.mdpi.com/1996-1944/13/24/5655/s1, Figure S1: The WAXD patterns of the L-PCL/PVC blends cooled from 100 °C down to 40 °C and kept at 40 °C for different times and the stretched amorphous L-PCL/PVC blend at 40 °C with a draw ratio of λ = 5 at a strain rate of 6 mm/min. Figure S2: The azimuthal profiles of the WAXD pattern shown in Figure 4a. Figure S3: The azimuthal profiles of the WAXD pattern shown in Figure 5. Figure S4: The WAXD patterns of the crystalline L-PCL/PVC blends taken during deformation at different draw ratios. Figure S5: Azimuthal profiles of the WAXD patterns shown in Figure 6.

## References

[1] Varga J., Karger-Kocsis J. (1993). Direct evidence of row-nucleated cylindritic crystallization in glass fiber-reinforced polypropylene composites. Polym. Bull..

[2] Varga J., Karger-Kocsis J. (1996). Rules of supermolecular structure formation in sheared isotactic polypropylene melts. J. Polym. Sci. Part B Polym. Phys..

[3] Sun X., Li H., Lieberwirth I., Yan S. (2007). α and β interfacial structures of the iPP/PET matrix/fiber systems. Macromolecules.

[4] Sun X., Li H., Wang J., Yan S. (2006). Shear-induced interfacial structure of isotactic polypropylene (iPP) in iPP/fiber composites. Macromolecules.

[5] Liu Q., Sun X., Li H., Yan S. (2013). Orientation-induced crystallization of isotactic polypropylene. Polymer.

[6] Sun X., Li H., Zhang X., Wang D., Schultz J.M., Yan S. (2010). Effect of matrix molecular mass on the crystallization of β-form isotactic polypropylene around an oriented polypropylene fiber. Macromolecules.

[7] Sun X., Li H., Zhang X., Wang J., Wang D., Yan S. (2006). Effect of fiber molecular weight on the interfacial morphology of iPP fiber/matrix single polymer composites. Macromolecules.

[8] Li H., Zhang X., Kuang X., Wang J., Wang D., Li L., Yan S. (2004). A scanning electron microscopy study on the morphologies of isotactic polypropylene induced by its own fibers. Macromolecules.

[9] Li H., Zhang X., Duan Y., Wang D., Li L., Yan S. (2004). Influence of crystallization temperature on the morphologies of isotactic polypropylene single-polymer composite. Polymer.

[10] Li H., Jiang S., Wang J., Wang D., Yan S. (2003). Optical microscopic study on the morphologies of isotactic polypropylene induced by its homogeneity fibers. Macromolecules.

[11] Petermann J., Gohil R.M. (1979). A new method for the preparation of high modulus thermoplastic films. J. Mater. Sci..

[12] Liu J., Zhao Q., Dong Y., Sun X., Hu Z., Dong H., Hu W., Yan S. (2020). Self-polarized poly (vinylidene fluoride) ultrathin film and its piezo/ferroelectric properties. ACS Appl. Mater. Interfaces.

[13] Hu J., Xin R., Hou C., Yan S. (2019). Preparation and self-repairing of highly oriented structures of ultrathin polymer films. Macromol. Chem. Phys..

[14] Loos J., Schauwienold A.M., Yan S., Petermann J., Kaminsky W. (1997). Crystallization of syndiotactic polypropylene (sPP) from oriented melts. Polym. Bull..

[15] Guadagno L., Naddeo C., Vittoria V. (2005). Temperature and orientation induced polymorphic behavior of syndiotactic polypropylene. Macromolecules.

[16] Kaito A., Iwakura Y., Hatakeyama K., Li Y. (2007). Organization of oriented lamellar structures in a miscible crystalline/crystalline polymer blend under uniaxial compression flow near the melting temperature. Macromolecules.

[17] Soto A., Voyiatzis G.A. (2002). Molecular orientation of poly (ethylene naphthalate)/poly (ethylene terephthalate) copolymers utilizing polarized raman spectra. Macromolecules.

[18] Soto A., Iconomopoulou S.M., Manikas A.C., Voyiatzis G.A. (2005). Molecular orientation of poly (ethylene terephthalate) and poly (butylene terephthalate) probed by polarized raman spectra: A parallel study. Appl. Spectrosc..

[19] Zheng Y., Zhang J., Sun X., Li H., Ren Z., Yan S. (2018). Enhanced αγ′ transition of poly (vinylidene fluoride) by step crystallization and subsequent annealing. Chin. J. Polym. Sci..

[20] Li Y., Kaito A., Horiuchi S. (2004). Biaxially oriented lamellar morphology formed by the confined crystallization of poly (1,4-butylene succinate) in the oriented blend with poly (vinylidene fluoride). Macromolecules.

[21] Kaito A. (2006). Unique orientation textures formed in miscible blends of poly (vinylidene fluoride) and poly [(R)-3-hydroxybutyrate]. Polymer.

[22] Lotz B., Wittmann J.C. (1987). Polyethylene-isotactic polypropylene epitaxy: Analysis of the diffraction patterns of oriented biphasic blends. J. Polym. Sci. Part B Polym. Phys..

[23] Nishio Y., Yamane T., Takahashi T. (1984). Crystallization behavior of high-density polyethylene in an oriented blend with polypropylene. J. Macromol. Sci. Part B Phys..

[24] Brinkmann M., Contal C., Kayunkid N., Tatjana D., Roland R. (2010). Highly oriented and nanotextured films of regioregular poly (3-hexylthiophene) grown by epitaxy on the nanostructured surface of an aromatic substrate. Macromolecules.

[25] Brinkmann M., Rannou P. (2007). Effect of molecular weight on the structure and morphology of oriented thin films of regioregular poly (3-hexylthiophene) grown by directional epitaxial solidification. Adv. Funct. Mater..

[26] Park Y.J., Kang S.J., Lotz B., Brinkmann M., Thierry A., Kim K.J., Park C. (2008). Ordered ferroelectric PVDF-TrFE thin films by high throughput epitaxy for nonvolatile polymer memory. Macromolecules.

[27] Hamidi-Sakr A., Schiefer D., Covindarassou S., Binek L., Sommer M., Brinkmann M. (2016). Highly oriented and crystalline films of a phenyl-substituted polythiophene prepared by epitaxy: Structural model and influence of molecular weight. Macromolecules.

[28] Li J., Xue M., Xue N., Li H., Zhang L., Ren Z., Yan S., Sun X. (2019). Highly anisotropic P3HT film fabricated via epitaxy on oriented polyethylene film and solvent vapor treatment. Langmuir.

[29] Hu J., Xin R., Hou C., Yan S., Liu J. (2019). Direct comparison of crystal nucleation activity of PCL on patterned substrates. Chin. J. Polym. Sci..

[30] Memon W.A., Li J., Fang Q., Ren Z., Yan S., Sun X. (2019). Synergistic effect of solvent and epitaxy on the formation of anisotropic structures of P3HT and P3HT/PCBM films. J. Phys. Chem. B.

[31] Guo Z., Xin R., Hu J., Li Y., Sun X., Yan S. (2019). Direct high-temperature form I crystallization of isotactic poly (1-butene) assisted by oriented isotactic polypropylene. Macromolecules.

[32] Guo Z., Li S., Liu X., Zhang J., Li H., Sun X., Ren Z., Yan S. (2018). Epitaxial crystallization of isotactic poly (methyl methacrylate) from different states on highly oriented polyethylene thin film. J. Phys. Chem. B.

[33] Xin R., Zhang J., Sun X., Li H., Qiu Z., Yan S. (2015). Epitaxial effects on polymer crystallization. Adv. Polym. Sci..

[34] Wang J., Liu Y., Li H., Yan S., Sun X., Tu D., Guo X., Ren Z. (2020). Enhanced charge transport and thermoelectric performance of P(NDI2OD-T2) by epitaxial crystallization on highly oriented polyethylene substrates. Mater. Chem. Front..

[35] Li Y., Guo Z., Xue M., Yan S. (2019). Epitaxial recrystallization of iPBu in form II on an oriented iPS film initially induced by oriented form I iPBu. Macromolecules.

[36] Dong H., Li H., Wang E., Wei Z., Xu W., Hu W., Yan S. (2008). Ordering rigid rod conjugated polymer molecules for high performance photoswitchers. Langmuir.

[37] Liu J., Li H., Yan S., Xiao Q., Petermann J. (2003). Surface induced crystallization of PCL on oriented PE substrates: Epitaxy and transcrystallization. Colloid Polym. Sci..

[38] Li L., Hu J., Li Y., Huang Q., Sun X., Yan S. (2020). Evidence for the soft and hard epitaxies of poly (L-lactic acid) on an oriented polyethylene substrate and their dependence on the crystallization temperature. Macromolecules.

[39] Guan G., Zhang J., Sun X., Li H., Yan S., Lotz B. (2018). Oriented overgrowths of poly (L-lactide) on oriented isotactic polypropylene: A sequence of soft and hard epitaxies. Macromol. Rapid Commun..

[40] Damman P., Coppée S., Geskin V.M., Lazzaroni R. (2002). What is the mechanism of oriented crystal growth on rubbed polymer substrates? Topography vs epitaxy. J. Am. Chem. Soc..

[41] Choi J., Cakmak M. (2016). Morphological evolution during thermal and strain induced crystallization in poly (ethylene terephthalate)/poly (ether imide) blend films. Polymer.

[42] Dikshit A.K., Kaito A. (2003). Crystallization and orientation behaviors in isotactic polystyrene and poly (2,6-dimethylphenylene oxide) blends. Polymer.

[43] Men Y., Strobl G. (2003). Critical strains in poly(ε-caprolactone) and blends with poly (vinyl methyl ether) and poly(styrene-co-acrylonitrile). Macromolecules.

[44] Park J.W., Tanaka T., Doi Y., Iwata T. (2010). Uniaxial drawing of poly[(R)-3-hydroxybutyrate]/cellulose acetate butyrate blends and their orientation behavior. Macromol. Biosci..

[45] Park J.W., Doi Y., Iwata T. (2005). Unique crystalline orientation of poly [(R)-3-hydroxybutyrate]/cellulose propionate blends under uniaxial drawing. Macromolecules.

[46] Mareau V.H., Prud’Homme R.E. (2003). Growth rates and morphologies of miscible PCL/PVC blend thin and thick films. Macromolecules.

[47] Morin D., Zhao Y., Prud’Homme R.E. (2001). Chain orientation resulting from the crystallization under strain of poly(ε-caprolactone) in miscible blends with poly (styrene-co-maleic anhydride). J. Appl. Polym. Sci..

[48] Coleman M.M., Zarian J. (1979). Fourier-transform infrared studies of polymer blends. II. Poly (ε-caprolactone)-poly (vinyl chloride) system. J. Polym. Sci. Polym. Phys. Ed..

[49] Hubbell D.S., Cooper S.L. (1977). Segmental orientation in blends of poly (ε-caprolactone) with poly (vinyl chloride) and nitrocellulose. J. Polym. Sci. Polym. Phys. Ed..

[50] Zhao Y., Keroack D., Prud’homme R. (1999). Crystallization under strain and resultant orientation of poly (ε-caprolactone) in miscible blends. Macromolecules.

[51] Zhang Y., Leblanc-Boily V., Zhao Y., Prud’homme R.E. (2005). Wide angle X-ray diffraction investigation of crystal orientation in miscible blend of poly (ε-caprolactone)/poly (vinyl chloride) crystallized under strain. Polymer.

[52] Zhang Y., Prud’Homme R.E. (2006). Crystallization of poly (ε-caprolactone)/ poly (vinyl chloride) miscible blends under strain: The role of molecular weight. Macromol. Rapid Commun..

[53] Bu X., Li H., Yan S. (2017). The propagation of crystal orientation in poly (ε-caprolactone)/poly (vinyl chloride) blend film after removal of induction layer. Colloid Polym. Sci..

[54] Jiang Z., Wang Y., Fu L., Whiteside B., Wyborn J., Norris K., Wu Z., Coates P., Men Y. (2013). Tensile deformation of oriented poly (ε-caprolactone) and its miscible blends with poly (vinyl methyl ether). Macromolecules.

[55] Kakudo M., Kasai N. (1972). X-ray Diffraction by Polymer.

[56] Li Y., You J. (2011). Micro-phase separation and crystallization behavior of amorphous oriented PLLA/PVAc blends during heat treatment under strain. Polymer.

[57] Nakagawa S., Kadena K., Ishizone T., Nojima S., Shimizu T., Yamaguchi K., Nakahama S. (2012). Crystallization behavior and crystal orientation of poly (ε-caprolactone) homopolymers confined in nanocylinders: Effects of nanocylinder dimension. Macromolecules.

[58] Judge J.T., Stein R.S. (1961). Growth of crystals from molten crosslinked oriented polyethylene. J. Appl. Phys..

[59] Jiang Z., Chen R., Lu Y., Whiteside B., Coates P., Wu Z., Men Y. (2017). Crystallization temperature dependence of cavitation and plastic flow in the tensile deformation of poly (ε-caprolactone). J. Phys. Chem. B.

