# Supplementary material for: Orientation of Poly(ε-caprolactone) in Its Poly(vinyl chloride) Blends Crystallized under Strain: The Role of Strain Rate
Source: Materials (Basel). 2020 Dec 11;13(24):5655. doi: 10.3390/ma13245655 (PMC7763942; doi:10.3390/ma13245655)
Supplement: Supplementary file 1 [file materials-13-05655-s001.pdf]

materials

Supplementary information

Orientation of Poly(ε-Caprolactone) in Its Poly(Vinyl Chloride) Blends Crystallized Under Strain: The Role of Strain Rate

Ruru Wan [1], Xiaoli Sun [1], Zhongjie Ren [1], Huihui Li [1,*] and Shouke Yan [1,2,*]

1 State Key Laboratory of Chemical Resource Engineering, Beijing University of Chemical Technology, Beijing 100029, China; 2018210183@mail.buct.edu.cn (R.W.); xiaolisun@mail.buct.edu.cn (X.S.); renzj@mail.buct.edu.cn (Z.R.)

2 Key Laboratory of Rubber-Plastics, Qingdao University of Science & Technology, Qingdao 266042, China.

* Correspondence: lihuihui@mail.buct.edu.cn (H.L.); skyan@mail.buct.edu.cn (S.Y.)

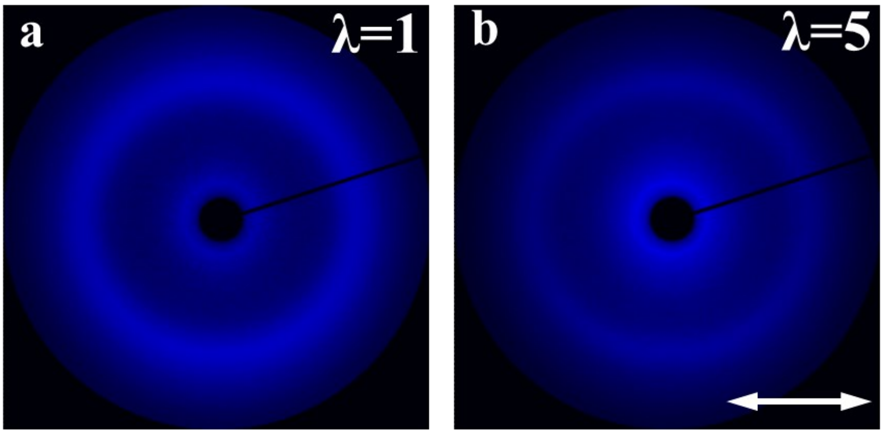

Figure S1. WAXD patterns of L-PCL/PVC blends film during stretching at 40 °C. (a) Unstretched film after erasing the thermal history and then rapidly cooled to 40 °C. (b) Stretched film after draw ratio just reached 6 at 40 °C. The arrow indicates the drawing direction.

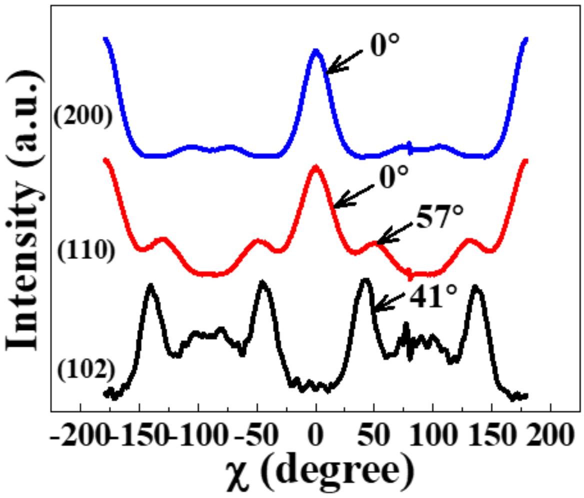

Figure S2. Azimuthal profiles of the WAXD pattern shown in Figure 4a of the main text.

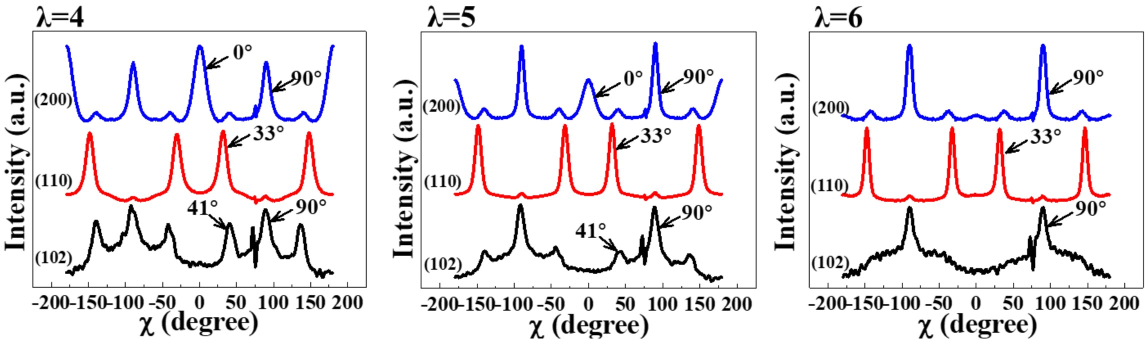

Figure S3. Azimuthal profiles of the WAXD patterns shown in Figure 5 of the main text.

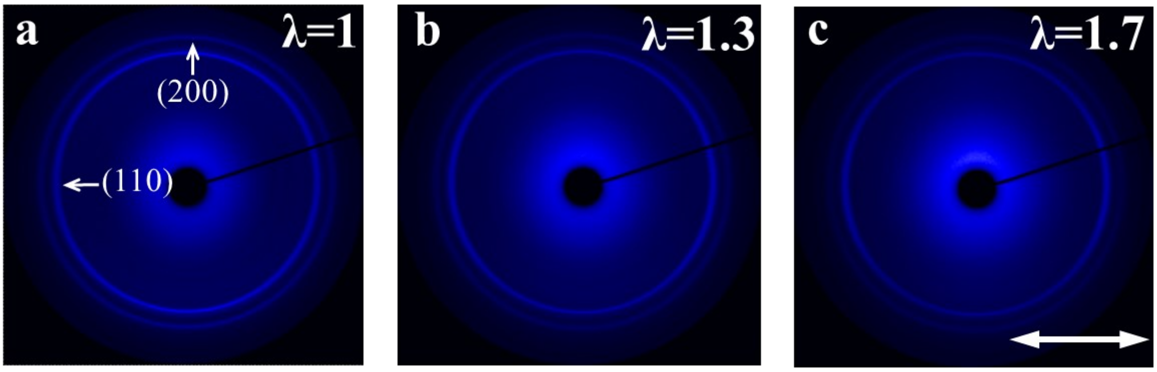

Figure S4. WAXD patterns of crystalline L-PCL/PVC (70/30) blend films drawn with a strain rate of 6 mm/min at room temperature to various draw ratios. (a) $\lambda = 1$, (b) $\lambda = 1.3$, (c) $\lambda = 1.7$. The draw direction is horizontal.

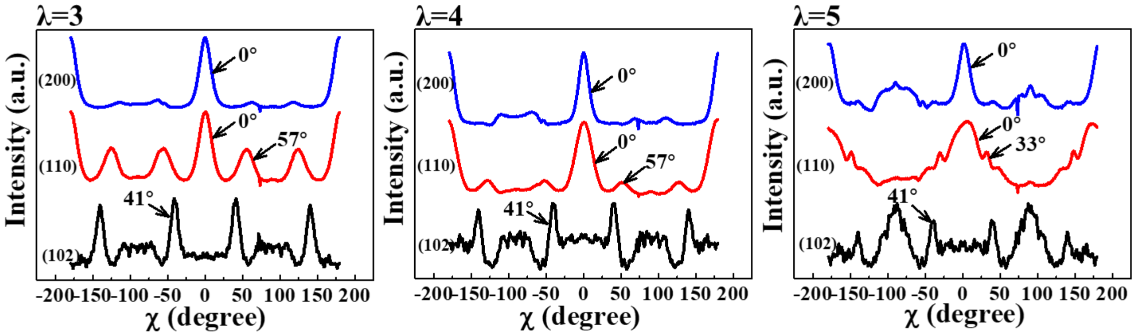

Figure S5. Azimuthal profiles of the WAXD patterns shown in Figure 6 of the main text.
